# Prognostic Value of Combined FDG PET and MRI Analysis of Cervical Cancer: A Systematic Review and Meta‐Analysis

**DOI:** 10.1002/jmrs.70103

**Published:** 2026-06-18

**Authors:** William Wei, Dominic Ku, Natalie Rutherford

**Affiliations:** ^1^ Austin Health Deakin University Heidelberg Victoria Australia; ^2^ Department of Radiology John Hunter Hospital Newcastle New South Wales Australia; ^3^ Department of Nuclear Medicine John Hunter Hospital Newcastle New South Wales Australia

## Abstract

**Introduction:**

Accurate risk stratification in cervical cancer remains challenging, and imaging biomarkers may add prognostic value beyond staging. This systematic review was conducted to evaluate the published data on the integrated use of 18F‐fluorodeoxyglucose (FDG) positron emission tomography (PET) and magnetic resonance imaging (MRI) in cervical cancer, with a specific analysis of the correlation between functional and anatomical imaging parameters and the identification of prognostic imaging markers.

**Methods:**

A comprehensive database search was conducted to identify studies that examined functional and anatomical imaging parameters using PET and MRI in the analysis of cervical cancer. A total of 328 articles were screened, and 107 full‐text articles were reviewed. 34 studies were ultimately included in the systematic review.

**Results:**

Thirty Four papers were reviewed for systematic review. Key functional and anatomical imaging parameters were analysed. 11 papers analysed PET standardised uptake value (SUV) and MRI apparent diffusion coefficient (ADC) correlations which were suitable for meta‐analysis. Moderate negative correlations between SUV and ADC values were statistically significant across multiple studies. Furthermore, metabolic tumour volume (MTV) and total lesion glycolysis (TLG) were shown to be statistically correlated with tumour grade, FIGO staging and survival outcomes (disease‐free survival, DFS, and overall survival, OS). Imaging parameters predictive of local recurrence, metastatic disease and survival outcomes were analysed.

**Conclusion:**

This systematic review of 34 studies on FDG PET and MRI in cervical cancer shows that imaging biomarkers predict recurrence, metastasis, and outcomes. Collectively, the literature supports the potential role of integrated PET/MRI biomarkers for improved prognostic stratification beyond conventional staging systems.

## Graphical abstract

This systematic review and meta‐analysis synthesised FDG PET/MRWE biomarkers in cervical cancer, including a pooled analysis of the association between metabolic activity (SUV‐based metrics) and tumour diffusion characteristics (ADC measures). Prognostic evidence from the included studies was also analysed to identify PET‐ and MRI‐based parameters, as well as combined indices, that are most consistently linked to survival and recurrence outcomes. Overall, the findings support multiparametric PET/MRI as a quantitative approach to risk stratification with potential to inform personalised treatment and response assessment.

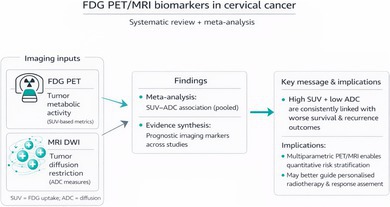

## 1 Introduction

Despite a global decline in cervical cancer incidence in women, largely attributed to improvements in hygiene, screening programs, and human papillomavirus vaccination efforts, cervical cancer remains the fourth most common cancer worldwide in terms of incidence and mortality [1]. Early‐stage localised cervical cancer has an excellent prognosis with a 5‐year survival rate of 91.5%, but this drops significantly to 16.5% for those with metastatic disease [2]. Therefore, precise and accurate diagnosis of cervical cancer is essential in tailoring treatment for patients.

Magnetic resonance imaging (MRI) is a well‐established modality for the assessment of cervical cancer. Its superior soft tissue resolution enables characterisation and staging of cervical cancers following the International Federation of Gynaecology and Obstetrics (FIGO) guidelines [3, 4]. A functional technique of MRI is the apparent diffusion coefficient (ADC), derived from diffusion‐weighted imaging (DWI) [5]. ADC reflects the diffusivity of water molecules, which is typically restricted in malignant tissues due to the high cell density, which impedes water diffusion [5]. Therefore, malignant tissue correlates with lower ADC values when compared with normal tissue [5, 6]. Lower ADC values correlate with more aggressive tumours and a poorer prognosis [5, 6].

However, MRI alone exhibits low sensitivity and specificity in detecting nodal involvement [7]. In early‐stage cancer, the sensitivity and specificity of lymph node metastasis were 52%–75% and 75%–91%, respectively [8]. Functional imaging modalities such as positron emission tomography (PET) using 18F‐fluorodeoxyglucose (FDG) are adjuncts in evaluating metastatic cervical cancer. As malignant tumours are metabolically active, they exhibit increased glucose metabolism facilitated by upregulation of membrane glucose transporters [9]. The standardised uptake value (SUV) measures the metabolic activity of the tumour, with higher SUV values indicating a more aggressive tumour [10].

Integrated PET/MRI systems offer potential for enhanced diagnostic accuracy by combining the functional imaging capabilities of PET with tissue characterisation provided by MRI. PET/MRI systems offer higher diagnostic accuracy for cervical cancer compared to PET/CT systems, MRI and CT alone, as well as higher detection rates of locoregional invasion [7].

Previous studies, and a subsequent systematic synthesis, have previously attempted to evaluate the utility of SUV and ADC in the diagnosis of cervical cancer, which have shown an inverse correlation between the two parameters [11, 12, 13, 14, 15, 16, 17, 18, 19, 20, 21, 22, 23]. The objective of this systematic review is therefore to evaluate existing studies and conduct a comprehensive statistical analysis to investigate the relationship between ADC values and SUV in cervical cancers.

## 2 Methods

### 2.1 Literature Search

**FIGURE 1:**
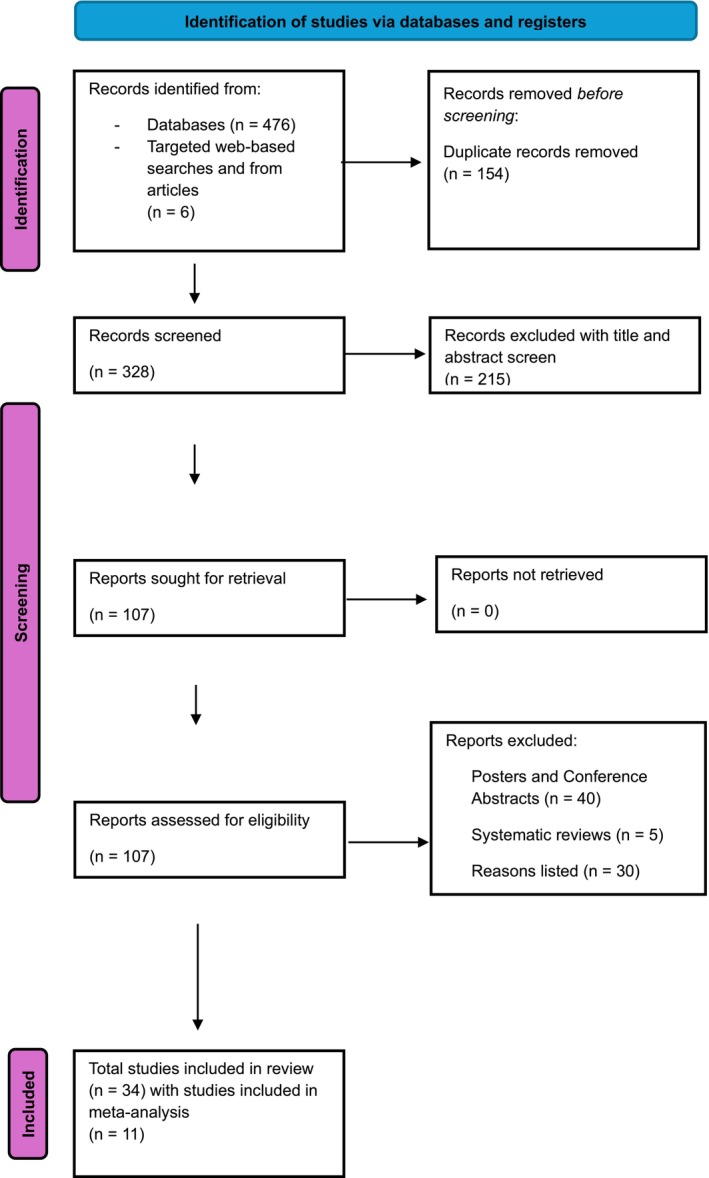
PRISMA 2020 flow diagram illustrating studies selected for the systematic review, created by author based on Page et al. [24].

A systematic literature search was conducted using Embase, Medline, and Cochrane to identify relevant studies published from January 1, 2000, to December 31, 2024. The search strategy was developed based on key terms related to cervical cancer, PET and MRI imaging, and their associated imaging biomarkers. Reproducible search strings for each database are provided in Appendix A. No language restrictions were applied during the search. All retrieved study titles and abstracts were screened to identify potentially relevant articles, and additional articles were identified through manual cross‐checking of the reference lists of included studies, as summarised in Figure 1.

The inclusion criteria were structured according to the PRISMA framework using the Patient/Intervention/Comparator/Outcome (PICO) model:

- Patient: Women diagnosed with new or recurrent uterine cervical cancer
- Intervention: Integrated imaging using PET and MRI, with specific analysis of functional biomarkers such as ADC and SUV.
- Comparator: No specific comparator was required for inclusion.
- Outcome: Primary outcomes included correlations between PET and MRI‐derived imaging parameters, as well as their prognostic value, particularly in relation to overall survival (OS), disease‐free survival (DFS). Other surrogates for DFS, including progression‐free survival (PFS) and event‐free survival (EFS), were also included.

Studies were excluded if they were not original research articles (e.g., reviews, editorials, conference abstracts), fell outside the scope of the study (e.g., focused on non‐cervical malignancies or unrelated imaging modalities), or did not report sufficient data on survival outcomes or correlations between imaging parameters. The studies included for review are summarised in Tables 1.1, 1.2, 1.3, 1.4.

**TABLE 1.1:** Summary of studies investigating SUV and ADC relationship.

Author	R vs P *	Year	No. of newly diagnosed patients/recurrent tumours	Stages					Indication (staging, restaging or treatment response)	Integrated, fused or separate PET/MRI	Conclusion
				1	2a	2b	3	4			
Xu et al. [25]	R	2020	95	66	12		17	0	Staging (predicting lymph node metastasis)	Integrated	SUVmax showed a negligible, non‐significant association with ADCmin (Spearman ρ = −0.039; *p* > 0.05).
Akkus Yildirim et al. [11]	R	2019	63	4	4	38	17	0	Staging	Separate	SUVmax correlated with tumour size and treatment response. ADCmean correlated with FIGO stage, size, and nodal metastasis. The two markers were inversely correlated (*r* = −0.44, *p* < 0.001).
Gong et al. [12]	R	2019	46	25	10	11	0	0	Staging	Integrated	SUVmax associated with tumour size, stage, and metastasis. ADCmin showed inverse trends. SUVmax and ADCmin were inversely correlated (*r* = −0.700, *p* < 0.001).
Li‐Ou et al. [26]	R	2019	54	17	8	11	15	3	Other	Integrated	Integrated PET/MR parameters demonstrated that glucose metabolism (SUVmax and SUVmean) significantly correlates with tumour hypoxia (*r* = 0.53) and perfusion (*r* = 0.54), but only weakly with diffusion (*r* = 0.29). Immunohistochemistry validated these findings, linking GLUT‐1 to SUV.
Yang et al. [13]	R	2019	72	6	21		35	10	Staging	Separate	PET/CT metabolic parameters predicted clinicopathology while MRI diffusion did not. Elevated SUVmax significantly associated with lymph node metastases (*p* = 0.002), distant metastases (*p* = 0.015), poor differentiation (*p* = 0.024), and advanced FIGO stage (*p* = 0.020). In contrast, ADCmin showed no significant correlations with pathological features, although a weak inverse correlation was found between SUVmax and ADCmin (*r* = −0.306, *p* = 0.036).
Floberg et al. [14]	R	2018	17	11	1	0	4	1	Staging	Integrated	A consistent significant inverse correlation between voxel SUVmax and ADCmean was found in squamous cell carcinomas (*r* = −0.35) and poorly differentiated tumours, whereas adenocarcinomas and well/moderately differentiated tumours showed no consistent relationship (*p* = 0.015).
Du et al. [15]	P	2018		10	7	10	15	2	Staging	Integrated	PET/MRI parameters predicted stage and histology. Advanced stage associated with elevated MTV, TLG and perfusion heterogeneity (*p* < 0.05). Higher tumour grade linked to increased MTV/TLG (*p* < 0.01) and reduced diffusion (AUC = 0.868). SUVmax moderately inversely correlated with ADCmin (ρ = −0.69), though most IVIM histogram parameters showed no metabolic correlation.
Meyer et al. [16]	R	2018	18	0	0	8	1	9	Staging	Integrated	PET/MRI parameters predicted metabolic‐diffusion associations. MTV and TLG significantly inversely correlated with whole‐lesion ADC histogram metrics (e.g., MTV vs. ADCmean = −0.546, *p* = 0.019; TLG vs. ADCmode *r* = −0.546, *p* = 0.019). In contrast, SUVmax and SUVmean showed no significant correlations with any ADC parameters.
Lai et al. [27]	R	2017	29 with 1 unstaged	8	2	7	10	1	Staging	Separate	MTV defined at 40%–45% SUVmax thresholds showed good agreement with the T2W gold standard (ICC = 0.81–0.83). In contrast, functional tumour volume derived from ADC maps showed poor concordance (ICC < 0.50) and significantly underestimated tumour volume compared to anatomical imaging.
Surov et al. [17]	P	2017	21	1	1	9	1	9	Staging	Integrated	PET/MRI parameters predicted N‐stage and proliferation. N‐positive tumours showed reduced ADCmin (*p* = 0.017). Higher Ki‐67 index associated with elevated SUVmax (ρ = 0.59) and reduced ADCmin (ρ = −0.48), but no significant correlation between SUVmax and ADCmin (ρ = −0.13, *p* = 0.59).
Pinker et al. [28]	P	2016	16	Not stated					Staging	Fused	Multiparametric PET/MRI confirmed tumour heterogeneity with weak voxel‐wise correlations between modalities (*r* = 0.05–0.22), although lesion‐based analysis showed significant correlations between metabolic activity and hypoxia (FDG vs. 18F‐fluoromisonidazole SUVmax, *p* = 0.04) and perfusion vs. cellularity (initial enhancement vs. ADC, *p* = 0.05).
Brandmaier et al. [18]	P	2015	14/17	Not stated					Staging and restaging	Integrated	Simultaneous PET/MRI parameters reflected tumour cellularity and recurrence status. SUVmax and ADCmin were significantly inversely correlated in both primary (*r* = −0.532, *p* = 0.05) and recurrent tumours (*r* = −0.747, *p* = 0.002). Additionally, primary tumours exhibited significantly higher SUVmax than recurrent lesions (*p* < 0.05), while ADC values did not significantly differ between the groups.
Grueneisen et al. [19]	P	2014	9/10	Not stated					Prospective, staging and restaging	Integrated	A strong inverse correlation between SUVmax and ADCmin was found in primary cervical cancers (*r* = −0.692, *p* < 0.001), but no significant correlation was found in recurrent lesions (*r* = −0.136, *p* = 0.451).
Sun et al. [20]	p	2014	35	0	4	13	17	1	Staging	Integrated	Hybrid PET/MRI demonstrated strong volume concordance. PET volumes defined at 35%–40% SUVmax matched T2W and DWI volumes (*p* < 0.05). While whole‐tumour SUVmax and ADCmin did not correlate (*r* = −0.074), intratumoral sub volume analysis revealed a significant inverse relationship (*p* < 0.001), with higher metabolic activity sub volumes showing progressively restricted diffusion.
Nakamura et al. [21]	P	2012	66	12	6	33	10	5	Treatment response	Fused	A strong inverse correlation was found between primary tumour SUVmax and ADCmin (*r* = −0.529, *p* < 0.001). High SUVmax significantly correlated with advanced FIGO stage, larger tumour size, and nodal metastasis, whereas ADCmin showed no significant correlation with these clinical features.
Ho et al. [22]	P	2009	33	17	4	10	1	1	Staging	Separate	In primary tumours, SUVmax and ADCmin were not correlated (*r* = −0.065, *p* = 0.72), whereas relative indices showed a significant inverse correlation (*r* = −0.526, *p* = 0.002).

Abbreviations: ADC, apparent diffusion coefficient; AUC, area under the curve; DWI, diffusion‐weighted imaging; FDG, fluorodeoxyglucose; FIGO, International Federation of Gynaecology and Obstetrics; GLUT‐1, glucose transporter 1; ICC, intraclass correlation coefficient; IVIM, intravoxel incoherent motion; MTV, metabolic tumour volume; PET/CT, positron emission tomography/computed tomography; PET/MRI, positron emission tomography/magnetic resonance imaging; SUV, standardised uptake value; T2W, T2‐weighted; TLG, total lesion glycolysis.

* R = Retrospective study, P = Prospective study.

**TABLE 1.2:** Summary of studies investigating survival and prognosis.

Author	R vs P *	Year	No. of newly diagnosed patients/recurrent tumours	Stages					Indication (staging, restaging or treatment response)	Integrated, fused or separate PET/MRI	Conclusion
				1	2a	2b	3	4			
Dhesi et al. [29]	R	2024	97	7	66		19	5	Staging and treatment response	Fused	T2WI and DWI demonstrated significantly higher specificity and accuracy compared to PET‐CT alone (89.2% and 77.0% vs. 43.2%, *p* < 0.00001). Combined PET/MR consensus yielded the highest sensitivity (78.3%) and significantly predicted PFS (*p* < 0.001), whereas PET‐CT alone did not (*p* = 0.164).
Skipar et al. [30]	P	2022	82	16	49		17		Treatment response	Fused	Higher HF/SUV50 score independently predicted worse DFS (HR 3.3, *p* = 0.001) and OS (HR 2.2, *p* = 0.025), outperforming single‐modality Ktrans, ADC, and SUV.
Shih et al. [31]	P	2021	54	10	12		21	11	Treatment response	Integrated	SUVmax independently predicted PFS (HR 4.57, *p* = 0.009), while ADCmin predicted OS (HR 0.02, *p* = 0.05). Advanced tumours showed lower ADCmin and higher MTV/TLG ratios. Predictors varied by stage: Stage I (MTV/ADCmin), Stage II (ADCmin/MTV), and Stage IV (SUVmax, TLG). Other metrics lost significance after multivariate adjustment.
Gao et al. [32]	P	2020	51	0	30		17	4	Treatment response	Integrated	Pre‐treatment and changes in PET/MRI parameters were able to identify high‐risk recurrence post‐treatment (pre‐TLG, Δ%D (slow diffusion coefficient) and lymph node status)
Akkus Yildirim et al. [11]	R	2019	63	4	4	38	17	0	Staging	Separate	Low ADCmean was an independent predictor of DFS (HR 2.1, *p* = 0.04) and OS (HR 2.94, *p* = 0.06). SUVmax was an independent predictor of DFS (HR 2.49, *p* = 0.03).
Ho et al. [33]	R	2019	93	29	7	30	21	6	Staging	Separate	Lower tumour ADC90th percentile independently associated with worse PFS (HR 2.55, *p* = 0.017).
Floberg et al. [14]	R	2018	17	11	1	0	4	1	Staging	Integrated	Global PET/MRI metrics (SUVmax, MTV, ADCmean) did not predict DFS. However, the voxel‐wise inverse correlation between SUV and ADC was a significant prognostic factor (*p* = 0.026). Tumours with high metabolism strongly matched restricted diffusion had significantly worse outcomes.
Ueno et al. [34]	R	2017	21	1	1	13	6	0	Treatment response	Separate	MTV and TLG (HR 4.73, *p* = 0.036) and lower 10th‐percentile ADC (HR 5.2, *p* = 0.048) independently predicted worse EFS on multivariate analysis.
Grueneisen et al. [35]	P	2015	27	13	4	3	5	2	Prospective, Staging	Integrated	Quantitative functional parameters significantly correlate with prognostic factors in primary cervical cancer, where poorly differentiated (G3) tumours exhibited significantly higher metabolic activity (SUVmax 16.7 vs. 10.1, *p* = 0.005) and restricted diffusion (ADCmin 0.51 vs. 0.67, *p* = 0.014) compared to well‐to‐moderately differentiated tumours
Micco et al. [36]	R	2014	49/17	18	2	18	5	6	Staging	Separate	Pre‐treatment quantitative ADCmean, along with MTV and TLG, were associated with high‐risk clinicopathological factors and predicted DFS and OS, while SUVmax and contrast enhanced MRI parameters demonstrated limited prognostic value.
Nakamura et al. [21]	P	2012	66	12	6	33	10	5	Treatment response	Fused	The combination of high SUVmax and low ADCmin was identified as a significant independent prognostic factor for both DFS (*p* = 0.0030) and OS (*p* = 0.0036). This combined phenotype outperformed either parameter alone, neither of which was independently prognostic in multivariate analysis.

Abbreviations: Δ%D, percentage change in diffusion; ADC, apparent diffusion coefficient; DFS, disease‐free survival; DWI, diffusion‐weighted imaging; G3, grade 3/poorly differentiated; HF/SUV50, hypoxic fraction/SUV50; HR, hazard ratio; Ktrans, volume transfer constant; MTV, metabolic tumour volume; PET‐CT, positron emission tomography‐computed tomography; PET/MRI, positron emission tomography/magnetic resonance imaging; SUV, standardised uptake value; T2WI, T2‐weighted imaging; TLG, total lesion glycolysis.

* *R* = Retrospective study, *P* = Prospective study.

**TABLE 1.3:** Summary of studies investigating treatment response.

Author	R vs P *	Year	No. of newly diagnosed patients/recurrent tumours	Stages					Indication (staging, restaging or treatment response)	Integrated, fused or separate PET/MRI	Conclusion
				1	2a	2b	3	4			
Dhesi et al. [29]	R	2024	97/	7	66		19	5	Staging and treatment response	Fused	T2WI/DWI scoring and consensus PET/MRI significantly outperformed PET‐CT alone in assessing treatment response (*p* < 0.01 for specificity and accuracy), primarily because PET‐CT had a low positive predictive value (28.8%) due to frequent misclassification of post‐treatment inflammation as residual disease.
Pasciuto et al. [37]	P	2023	88	3	9	63	13	0	Treatment response	Fused	High final DWI signal (OR 4.04) and low ADCmean (OR 2.47) predicted partial response (AUC 0.81, *p* < 0.0001). Greater baseline‐to‐final SUVmax decrease independently predicted complete response (AUC 0.84, *p* = 0.004).
Vojtisek et al. [38]	P	2021	66	4	1	55	5	1	Treatment response	Fused	On week‐5 PET/MRI, non‐responders displayed higher mid‐treatment MTV, size, and TLG parameters (AUC 0.71–0.74, all *p* < 0.05) and smaller Δ%SUVmax (AUC 0.69, *p* = 0.021).
Gao et al. [32]	P	2020	51	0	30		17	4	Treatment response	Integrated	Lower Δ%D (AUC 0.735, *p* = 0.013) and Δ%F (AUC 0.859, *p* = 0.006) predicted poor early shrinkage. A combined model achieved AUC 0.901 (sensitivity 84%, specificity 94%).
Kalash et al. [39]	R	2018	244	42	10	131	61	0	Treatment Response	Separate	For patients with an incomplete metabolic response on PET/CT (20% of cohort), the addition of MRI effectively discriminated between true residual disease and inflammation, with positive DWI findings significantly predicting local recurrence (81.8% vs. 12.5%) and inferior overall survival (36% vs. 83%, *p* = 0.049).
Mongula et al. [40]	P	2018	10	1	0	8	0	1	Treatment response	Integrated	At 11 weeks post‐radiotherapy, PET‐MRI outperformed MRI for residual tumour detection (AUC 0.83 vs. 0.55, *p* < 0.01). PET alone achieved AUC 0.95. PET‐MRI increased diagnostic confidence (80%–90%), changed opinion (70%), and altered management (50%).
Sarabhai et al. [41]	P	2018	8	3	1	3	1	0	Treatment response	Integrated	In responders, tumour size, SUVmax, and perfusion (Ktrans, Kep, iAUC) decreased (39%–64%), while ADC increased (38%–39%). The single non‐responder showed minor size/SUVmax reductions, but increases in Ktrans, Kep, and iAUC (15%–22%), and ADC (24%–35%)
Ueno et al. [34]	R	2017	21	1	1	13	6	0	Treatment response	Separate	Non‐responders had higher pre‐treatment MTV (78.5 vs. 34.8 mL, *p* = 0.04) and TLG (68.8 vs. 24.2 g, *p* = 0.01). SUV and ADC metrics did not differ significantly.

Abbreviations: Δ%D, percentage change in diffusion; Δ%F, percentage change in diffusion fraction; ADC, apparent diffusion coefficient; AUC, area under the curve; DWI, diffusion‐weighted imaging; iAUC, initial area under the curve; Kep, efflux rate constant; Ktrans, volume transfer constant; MRI, magnetic resonance imaging; MTV, metabolic tumour volume; OR, odds ratio; PET/CT, positron emission tomography/computed tomography; PET/MRI, positron emission tomography/magnetic resonance imaging; SUV, standardised uptake value; T2WI, T2‐weighted imaging; TLG, total lesion glycolysis.

* *R* = Retrospective study, *P* = Prospective study.

**TABLE 1.4:** Summary of studies investigating metastatic potential.

Author	R vs P *	Year	No. of newly diagnosed patients/recurrent tumours	Stages					Indication (staging, restaging or treatment response)	Integrated, fused or separate PET/MRI	Conclusion
				1	2a	2b	3	4			
Esfahani et al. [42]	R	2021	14	4	0	3	0	7	Staging	Integrated	Metastatic disease demonstrated higher metabolic burden and heterogeneity. MTV and TLG were significantly elevated in metastases, with PET/CT MTV achieving the strongest discrimination (AUC 0.98), followed by PET/CT TLG (0.92) and PET/MRI metrics (AUC 0.82–0.85). Textural features (T2 compactness, ADC entropy) also significantly differentiated metastatic status (AUC 0.86, *p* < 0.05).
Steiner et al. [43]	R	2021	33	No FIGO Gr 1 = 5 Gr 2 = 15 Gr 3 = 13					Staging	Integrated	The SUVmax/ADCmean ratio predicted pelvic nodal metastasis (median 13.4 vs. 8.9, *p* = 0.032; AUC 0.81), outperforming SUVmax (*p* = 0.24) and ADCmean (*p* = 0.20) alone.
Xu et al. [44]	P	2021	90	84	6		0	0	Staging (predicting LV space invasion)	Integrated	In patients without nodal metastasis, LVSI‐positive tumours exhibited higher TLG, MTV, SUVmax/mean, and lower Dmin and ADCmin (AUCs 0.65–0.78; all *p* < 0.05). A combined TLG and Dmin model provided the strongest prediction (AUC 0.86).
Xu et al. [25]	R	2020	95	66	12		17	0	Staging (predicting lymph node metastasis)	Integrated	Pelvic nodal metastasis associated with higher TLG (AUC 0.692, *p* = 0.009), SUVmax (AUC 0.654, *p* = 0.023), and lower Dmin (AUC 0.681, *p* = 0.007). A combined model (TLG, Dmin, PET status) yielded AUC 0.913 (*p* < 0.001). In PET‐negative patients, TLG independently predicted occult metastasis (AUC 0.855, *p* < 0.001).
Gong et al. [12]	R	2019	46	25	10	11	0	0	Staging	Integrated	Primary‐tumour SUVmax associated with lymph node metastasis (AUC 0.681, *p* = 0.040) whereas ADCmin was non‐significant (AUC 0.643, *p* = 0.11).
Yang et al. [13]	R	2019	72	6	21		35	10	Staging	Separate	Higher primary‐tumour SUVmax and SUVmean were associated with lymph node (*p* = 0.002 and *p* = 0.005) and distant metastases (*p* = 0.015 and *p* = 0.036). In contrast, ADCmin showed no significant difference between metastatic and non‐metastatic disease for either nodal (*p* = 0.54) or distant involvement (*p* = 0.30).
Grueneisen et al. [45]	P	2014	17/5	Not stated					Staging	Integrated	Adding DWI to whole‐body PET/MRI offered no significant diagnostic benefit for staging pelvic malignancies, suggesting that DWI can be omitted to reduce scan time without compromising the detection of metastases.
Kitajima et al. [46]	R	2014	30	14	3	8	4	1	Staging	Fused retrospectively	Fused PET/MRI detected pelvic nodal metastasis with 92.3% sensitivity and 88.2% specificity. This performance matched contrast‐enhanced PET/CT and outperformed MRI alone (sensitivity 69.2%, specificity 100%) and side‐by‐side PET/CT plus MRI (sensitivity 84.6%, specificity 94.1%).

Abbreviations: ADC, apparent diffusion coefficient; AUC, area under the curve; Dmin, minimum diffusion coefficient; DWI, diffusion‐weighted imaging; FIGO, International Federation of Gynaecology and Obstetrics; Gr, grade; LVSI, lymphovascular space invasion; MTV, metabolic tumour volume; PET/CT, positron emission tomography/computed tomography; PET/MRI, positron emission tomography/magnetic resonance imaging; SUV, standardised uptake value; T2, T2‐weighted; TLG, total lesion glycolysis.

* *R* = Retrospective study, *P* = Prospective study.

When constructing the analytical tables, we categorised the literature based on whether the PET/MRI systems were integrated, fused, or separate. Integrated systems use a single hybrid scanner that simultaneously acquires PET and MRI data within the same gantry, ensuring perfect spatial and temporal alignment. Fused PET/MRI refers to a software‐driven approach where data from two distinct scans (or sequential acquisitions) are digitally overlaid or registered post‐acquisition to create a combined image. Conversely, separate PET/MRI denotes a traditional workflow in which the two scans are performed on separate machines at different times, with the resulting images viewed independently or compared side by side without technical integration.

Two reviewers carried out independent screenings and selections for the systematic reviews and meta‐analyses studies. Any disagreements were resolved through discussion and consensus. Risk of bias was assessed independently by the two reviewers using the QUIPS tool, with disagreements resolved by consensus. Full domain‐level ratings are provided in Table S1.

### 2.2 Statistical Analysis

**FIGURE 2:**
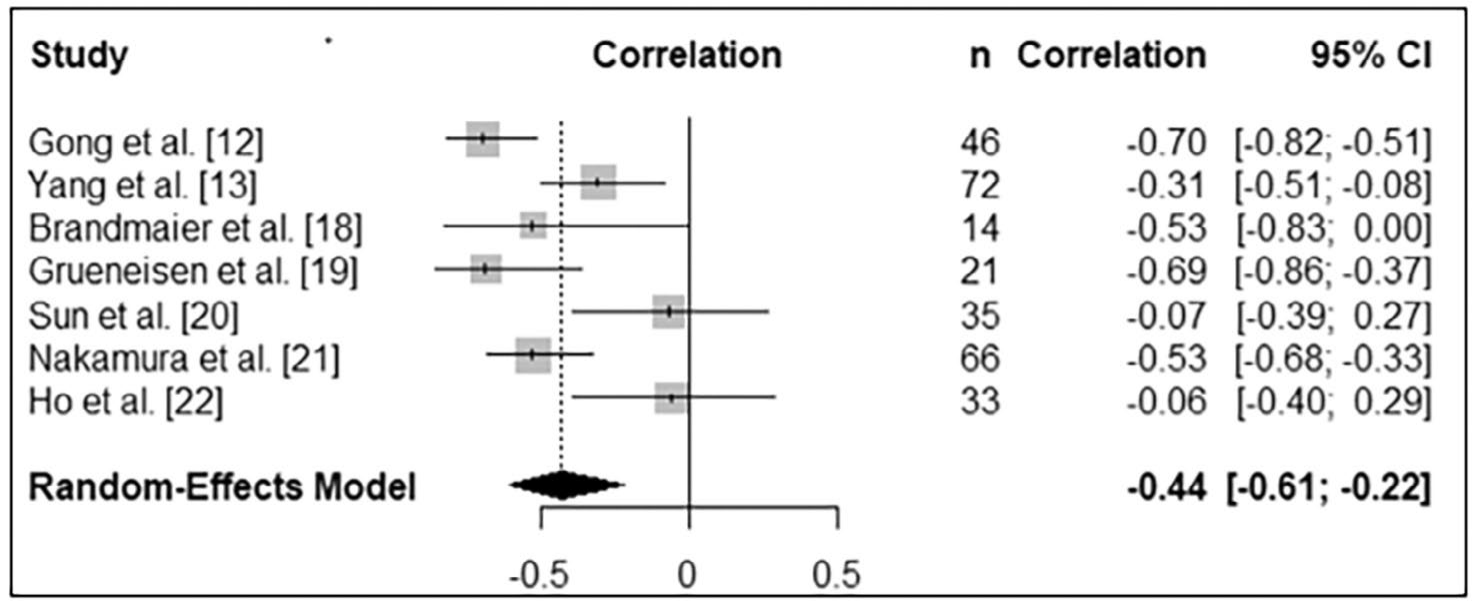
Pooled correlation of ADCmin and SUVmax of studies utilising Pearson's Correlation Coefficient.

A meta‐analysis of studies reporting Pearson correlation coefficients (r) for the relationship between ADCmin and SUVmax was conducted using the metacor function from the meta package in R (version 4.5.0, R Core Team, 2025). Reported Pearson's r values from eligible studies were transformed to Fisher's z scores internally by the function to stabilise variance and permit appropriate aggregation across studies. The studies are outlined in Figure 2.

**FIGURE 3:**
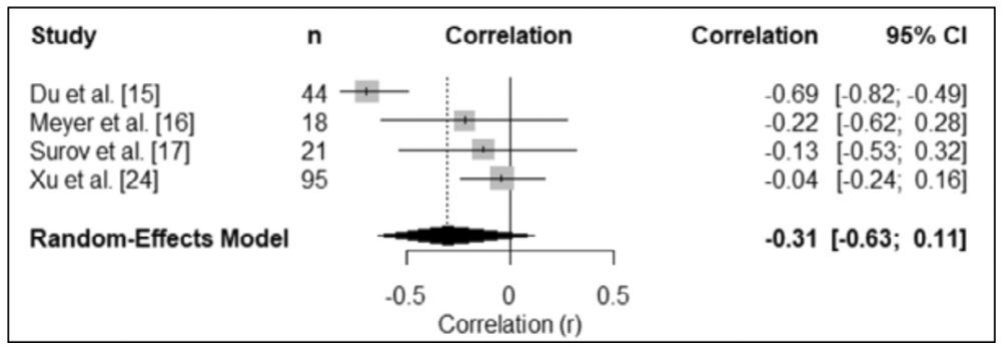
Pooled correlation of ADCmin and SUVmax of studies utilising Spearman's Correlation Coefficient.

For studies that reported Spearman's rank correlation coefficients (ρ), meta‐analysis was again conducted using the metacor function from the meta package in R. Effect sizes were converted to Fisher's *z* values to facilitate meta‐analytic pooling. While the transformation is formally derived for Pearson's r, it is commonly applied to Spearman's *ρ* in meta‐analyses due to its bounded distribution and approximately normal behaviour in large samples. The studies are outlined in Figure 3.

Reporting bias was not formally assessed in the meta‐analyses because fewer than 10 studies were available per analysis.

ADCmin was selected as the primary diffusion biomarker because it captures the most cellular, solid components of the tumour and reflects regions of highest cellular density. Focusing on ADCmin also mitigates the confounding dilution effect introduced by intratumoral necrosis or oedema, which can artificially elevate ADCmean values.

## 3 Results

Across the included studies, there was a consistent inverse relationship between FDG uptake and diffusion [11, 12, 13, 16, 18, 19, 21, 22]. Tumours with higher SUV (especially SUVmax) generally showed lower ADC values, reflecting higher cellularity and more aggressive microstructure [12, 13, 14, 15, 16, 17, 18, 21, 22]. Higher SUV and larger MTV were observed in higher FIGO stages, larger primary tumours, poorer differentiation, higher Ki‐67, and nodal involvement [11, 12, 13, 15, 17, 21]. Advanced multiparametric work (Intravoxel Incoherent Motion (IVIM), histogram and radiomic features) showed that PET metabolism, diffusion, perfusion and susceptibility all capture overlapping but non‐identical aspects of tumour biology, with only moderate correlations between parameters [16, 26, 28]. Volume concordance studies further highlighted that T2, DWI/ADC and PET MTV only partially overlap, reinforcing the idea that each sequence delineates different components of the tumour and that PET/MRI offers a richer composite picture than any single modality [20, 27].

Across the reviewed studies, a consistent prognostic pattern emerged: high baseline metabolic activity (elevated SUVmax, MTV, or TLG) paired with restricted diffusion (low mean, minimum, or percentile ADC values) was uniformly associated with poorer DFS, PFS, and OS [11, 14, 21, 31, 33, 34, 36]. Multivariable analyses frequently demonstrated that these parameters remain independent predictors of survival after adjusting for stage and clinical factors [11, 21, 30, 31, 33, 34]. Notably, combined indices, such as SUV/ADC ratios and integrated PET–IVIM models, often outperformed single metrics, suggesting that the intersection of high metabolic burden and restricted diffusion provides the most robust identification of high‐risk patients [14, 21, 30, 31]. Collectively, these findings support the utility of multiparametric PET/MRI as a quantitative risk‐stratification tool capable of refining prognostication beyond conventional staging [14, 29, 30, 31, 36].

Evidence from treatment‐response studies indicates that PET/MRI is valuable at both baseline and during chemoradiation for identifying patients at high risk of treatment failure [32, 34, 38]. Non‐responders frequently exhibited a pre‐treatment phenotype of high metabolic volume (MTV/TLG) and restricted diffusion [37, 38]. Longitudinal analyses, including serial PET/MRI and PET–IVIM, demonstrated that early quantitative changes in metabolism and diffusion, such as percentage reductions in SUV, TLG, or IVIM parameters, effectively tracked tumour regression and predicted recurrence [32, 37, 38, 41]. Furthermore, qualitative and semi‐quantitative response assessments on T2WI, DWI, and PET stratified prognosis, with imaging‐defined complete response linked to superior PFS and OS compared with residual abnormality [29, 39].

Regarding metastatic potential, multiparametric PET/MRI models showed improved prediction of microscopic spread [25, 43, 44]. Elevated primary tumour metabolic markers (SUVmax, MTV, TLG) and adverse diffusion/IVIM features were consistently associated with pathologically confirmed nodal metastases and lymphovascular space invasion (LVSI), even in patients with clinically node‐negative disease [12, 13, 25, 44]. Composite models integrating metabolic volume (particularly TLG) with diffusion parameters (e.g., Dmin) achieved high discrimination for occult spread, outperforming traditional imaging criteria [25, 43, 44]. Integrated PET/MRI demonstrated improved staging accuracy compared to MRI alone, while emerging radiomics data suggest that textural features from both modalities can further differentiate local from metastatic phenotypes (42 {Grueneisen, 2014 #40)}.

A random‐effects meta‐analysis of seven studies demonstrated a statistically significant, moderate negative correlation, with a pooled Pearson coefficient of −0.44 (95% CI: −0.62 to −0.21). Substantial heterogeneity was observed (I^2^ = 73%, Q = 22.10, *p* < 0.001), indicating significant variation in effect sizes across the studies.

A random‐effects meta‐analysis of four studies yielded a pooled Spearman correlation coefficient of −0.31 (95% CI: −0.61 to 0.07). Unlike the Pearson analysis, this correlation was not statistically significant. Substantial heterogeneity was observed (I^2^ = 84%, Q = 18.94, *p* < 0.001), driven largely by the discrepancy between the strong negative correlation in Du et al. (ρ = −0.69) and the negligible correlation in Xu et al. (ρ = −0.04).

### 3.1 Risk of Bias in Included Studies

Overall, the risk of bias across included studies was predominantly moderate, with several studies assessed as having high risk of bias in one or more domains. The most frequent sources of bias related to confounding and statistical analysis and reporting, which reflects limited adjustment for key clinical and pathological variables and variability in analytical transparency across studies. Bias related to study participation and attrition was generally low to moderate, although some studies showed a higher risk due to selective cohorts and incomplete follow‐up reporting. Assessment of prognostic factor measurement and outcome measurement was mostly moderate, with occasional concerns arising from heterogeneous imaging protocols, variable parameter definitions, and inconsistent outcome ascertainment. Full domain‐level QUIPS assessments for each included study are provided in the Supporting Information material.

## 4 Discussion

### 4.1 Current Utility of FDG PET and MRI

Pelvic MRI and FDG PET/CT are standard modalities for evaluating primary gynaecological malignancies and metastases, respectively. MRI provides excellent soft tissue delineation, allowing for accurate local staging, while FDG PET/CT has become indispensable in identifying nodal and distant metastases [47]. In the systematic review by Nguyen et al., they reported that fused FDG PET/MRI improves tumour staging and pelvic nodal assessment compared when compared to FDG PET/CT [23]. Multiple authors specifically demonstrate improved diagnostic accuracy in cervical cancer [19, 20, 22, 29, 40, 43, 46]. Simultaneous, also known as integrated PET/MRI, reduces motion and misregistration artefacts. It also provides the additional benefit of reduced radiation exposure, which is relevant in young female patients requiring regular follow‐up for cervical cancer [23, 48].

### 4.2 Rationale of Integrated FDG PET/MRI Analysis

Integrated FDG PET/MRI provides an opportunity to analyse functional and anatomical imaging parameters simultaneously [12, 14, 29, 30, 31, 43, 45]. FDG PET findings have been correlated with histopathological findings in cervical cancer such as increased GLUT‐1 and VEGF expression, in keeping with increased metabolism [26]. Conversely, MRI determines the depth of tumour involvement and parametrial involvement [46, 49, 50], and ADC provides further information on tumour cellularity [19].

The functional and anatomical information derived from FDG PET and MRI correlates across multiple studies. These papers demonstrate a moderate negative correlation with statistical significance between SUV values and ADC minimum values [13, 15, 18, 21]. This was shown to correlate with tumour size and pathological grade, with poorly differentiated tumours showing higher SUV values and lower ADC minimum values compared to well‐differentiated cervical tumours [12, 13, 15, 17, 36, 51]. These findings are crucial in establishing SUV and ADC as independent biomarkers that could predict tumour aggressiveness.

### 4.3 SUVmax And ADCmin Correlation

As shown in Figure 2, our pooled Pearson correlation analysis demonstrated a statistically significant moderate negative correlation between SUVmax and ADCmin. This finding indicates that tumours with higher metabolic activity tend to exhibit increased cellularity and restricted diffusion. These results are consistent with biological understanding, as more aggressive tumours often display increased glycolytic activity and higher cell density. In contrast, as shown in Figure 3, the pooled analysis of Spearman's correlations displayed a moderate, yet statistically non‐significant, negative association. The wider confidence interval and lack of statistical significance in this non‐parametric analysis underscore the variability in study methodologies and patient populations and the limited number of available studies reporting non‐parametric correlation data.

Substantial heterogeneity was observed in both correlation analyses, with an I^2^ = 73% in the Pearson correlation analysis and I^2^ = 84% in the Spearman correlation analysis. This significant variability can be attributed to several factors across the included studies, such as differences in imaging protocols, region of interest placement methods, b‐values used for ADC measurement, and diverse patient characteristics. Despite this heterogeneity, the overall analysis confirms a meaningful inverse relationship between SUVmax and ADCmin values. However, the consistent variability across studies highlights a need for standardised imaging methodologies and larger prospective trials. Such efforts are important for validating these findings and defining their prognostic utility.

For the Spearman's analysis, Fisher's Z‐transformation was employed to enable the pooling of *p*‐values under an assumption of normality. This allowed for the application of established meta‐analytic methods, stabilisation of variance, and generating more reliable pooled results, while maintaining consistency across both Pearson and Spearman analyses. However, it is important to acknowledge that this approach introduces parametric assumptions to a fundamentally non‐parametric context measure. Future meta‐analyses could consider alternative methods, such as rank‐based meta‐analysis, or separate reporting of Spearman's correlations to circumvent these assumptions.

### 4.4 Prognosticating Potential of SUV and ADC Values

There is strong agreement that combining metabolic and functional data outperforms single‐modality assessment for prognosis. Akkus et al. and Nakamura et al. both established that whilst SUVmax and ADC are individually prognostic, their combined use provides independent predictive values for DFS and OS [11, 21]. This reflects PET and MRI interrogate distinct but complementary biological axes.

However, relying solely on SUVmax is increasingly viewed as an oversimplification that misses intratumoral heterogeneity, as SUVmax captures only the hottest voxel and can miss the overall disease burden, which may be related to clinical outcomes. Micco et al. advocated for volume‐based markers such as MTV and TLG to improve risk stratification [36]. Shih et al. introduced a critical nuance, finding that the dominant prognostic marker shifts with disease stage [31]. This stage‐dependence implies that the biology underpinning failure is not uniform across the disease spectrum, and that no single PET or MRI metric is likely to perform optimally in all settings. They noted that SUVmax was predictive for PFS, whereas ADCmin were key drivers for OS [31]. This utility of ADC is supported by Dhesi et al., who reported that MRI could effectively differentiate aggressive tumours in treatment non‐responders [29]. Collectively, this suggests that metabolic activity may drive early recurrence, whilst cellular density and perhaps resistance to therapy may dictate long‐term survival.

Furthermore, the uncoupling of SUV and ADC provides unique biological insights. Skipar et al. utilised a multimodal score incorporating hypoxia (HF/SUV50), demonstrating that this composite biomarker can outperform standard metrics [30]. Accordingly, interpreting where SUV and ADC decouple (and combining them with complementary markers such as hypoxia surrogates or radiomics) may better separate viable tumour from treatment‐related or stromal change than either metric alone. In other cancers, such as necrotic lung carcinomas, discordant areas often indicate fibrosis (dense, but metabolically inactive) or inflammatory rims (metabolically active, but often oedematous) rather than viable tumour, underscoring the limitations of simple correlation models for complex tissue characterisation [52].

Finally, the choice of metric remains a source of variability. Whilst Nakamura et al. and Shih et al. focused on ADCmin, Ho et al. utilised volumetric ADC percentiles [21, 22, 31]. Studies in endometrial cancers suggest that ADCmin is susceptible to single‐pixel noise and that histogram analyses or advanced diffusion models are more stable predictors of prognosis [53]. For example, Gao et al. suggested that IVIM enables separation of true molecular diffusion from microcapillary perfusion, which may offer more stable prognostic prediction [32].

### 4.5 Treatment Response Using SUV and ADC

Response prediction differs from survival prognostication by targeting the early biological effects of chemoradiotherapy. Across the reviewed literature, temporal dynamics in imaging markers consistently outperform static baseline measures for predicting local control, because baseline scans describe initial phenotype but do not reliably capture radiosensitivity or early treatment reaction.

Several studies show that the percentage change in metabolic metrics is strongly associated with response. Sarabhai et al. and Mongula et al. demonstrated that the changes in metabolic activity (SUVmax, TLG) predict response [40, 41]. However, Vojtisek et al. cautioned that relying on simple maximum values requires nuance; while mid‐SUVmax and Δ%SUVmax were useful, they did not always clearly distinguish metabolic responders [38].

Complementing metabolic change, diffusion‐based shifts provide a structural correlate of treatment effect. Pasciuto et al. observed that post‐treatment patterns, such as high DWI signal combined with low ADCmean post‐treatment, were associated with residual tumour, whereas higher ADC values predicted a complete pathological response [37].

Importantly, there is evidence to support combining these modalities. Sarabhai et al. noted that combining dynamic shifts of both SUV and ADC yielded higher predictive accuracy, consistent with response requiring both metabolic cessation and microstructural change [41]. Gao et al. similarly showed that integrating PET with MRI data improved prediction of early tumour shrinkage [32].

The timing of these assessments is crucial for clinical utility. Vojtisek et al. utilised mid‐treatment PET/MRI and found on‐treatment metabolic markers were valuable for identifying non‐responders early enough to facilitate adaptive therapy [38]. While Pasciuto et al. suggest that baseline and post‐treatment imaging may suffice, the broader consensus supports using temporal dynamics (Δ%SUVmax and Δ%ADC) as early indicators of treatment efficacy [37].

A significant divergence from standard prognostic modelling is the shift toward texture analysis and radiomic approaches, which may capture complexities of radiation response. Ueno et al. supported this by finding that ADC10% added predictive value beyond basic metrics [34]. Kalash et al. and Pasciuto et al. utilised radiomics‐derived heterogeneity measures (e.g., Entropy, Dissimilarity) correlates with poor treatment response [39]. Together, these data suggest spatial heterogeneity may be a key determinant of treatment response.

### 4.6 Predictive Value of Additional Metabolic Parameters (MTV, TLG)

Several included studies highlight MTV and TLG as PET biomarkers that may better represent whole‐tumour metabolic burden (and heterogeneity) than SUVmax. Micco et al. advocated for these volume‐based markers over SUVmax, reporting stronger predictive power and clinical relevance for outcome stratification which is plausible in tumours with mixed viable, hypoxic/necrotic, and inflammatory subregions where SUVmax can be skewed by focal hotspots and technical artefact [36].

Timing also appears important. Vojtisek et al. found that while standard SUV metrics failed to distinguish metabolic responders during therapy, mid‐treatment MTV and TLG demonstrated significant predictive power [38]. Gao et al. incorporated pre‐treatment TLG into a combined model to predict early tumour shrinkage [32]. These mid‐treatment values (and treatment‐induced change) may function as an in vivo assay of radiosensitivity and residual viable disease, potentially explaining why some cohorts show stronger prognostic associations at interim timepoints than at baseline [32, 34, 38]. Ueno et al. reinforced this, reporting significantly higher MTV and TLG in non‐responders, suggesting that total metabolic burden correlates better with resistance than simple intensity metrics [34]. Correlations with tumour size and inverse relationships with diffusion metrics in some studies also support that these PET volumetric measures capture overlapping but non‐identical biology to MRI diffusion, metabolic burden versus cellularity, arguing for complementary multiparametric models rather than single markers [32, 34, 38].

Translation is limited by technical variability. MTV/TLG depend on segmentation method (thresholds, gradients, inclusion of necrosis/nodes), acquisition/reconstruction, uptake time, and inconsistent definitions of mid‐treatment, reducing inter‐study comparability and the portability of cut‐offs [16, 18, 20, 28, 30, 34]. TLG also embeds SUVmean and is often collinear with SUV/volume metrics; combined with small cohorts, this increases overfitting risk without pre‐specified models and external validation [15, 19, 33, 42]. Future studies should standardise delineation and timepoints, report incremental value over clinical factors and conventional PET/MRI parameters, and validate clinically actionable thresholds before MTV/TLG are used to guide response‐adaptive decisions [15, 16, 19, 28, 30, 33, 34, 42].

### 4.7 PET/MRI Parameters Predictive of Metastatic Potential

Metastatic spread remains difficult to predict because it reflects whole‐tumour disease load and biological capacity for dissemination, not just what is anatomically visible. In that context, volumetric PET measures (MTV/TLG) are conceptually compelling because they approximate total viable metabolic burden, which is more plausibly linked to microscopic escape than a single highest‐uptake voxel that can be influenced by noise and partial‐volume effects [41]. This also explains why metabolic volume can add value even when nodal morphology appears normal, acting as a proxy for subclinical disease burden that conventional imaging can miss [41].

Diffusion contributes complementary biology, but on its own, it is a limited metastasis discriminator because restricted diffusion is not specific and can be confounded by reactive or inflammatory changes [12, 13]. Its value is better framed as a modifier of metabolic risk: when high metabolic activity co‐exists with restricted diffusion, the combined signature may reflect a densely cellular, metabolically active phenotype aligned with aggressive behaviour and propensity for spread [42]. Overall, the literature supports prioritising integrated multiparametric models (such as SUV/ADC ratios) over single metrics for metastatic risk stratification [42].

### 4.8 Limitations

The included studies are heterogeneous in study design, imaging protocols and reported imaging parameters. For example, this analysis found that 19 of the 34 studies utilised integrated PET/MRI, with implications for spatial co‐registration and voxel‐level data correlation. Many studies lack multivariate analysis, failing to adjust for clinical confounders like FIGO stage or nodal status. This makes it difficult to determine if an imaging biomarker's prognostic value is truly independent or merely reflects its association with these established clinical factors, thereby limiting conclusions on its unique additive clinical utility. Furthermore, inconsistent definitions of survival metrics across studies complicate outcome comparisons and the synthesis of research findings. This variability hinders direct comparison of a biomarker's prognostic strength, makes meta‐analyses challenging, and obscures clear clinical interpretation, slowing the adoption of potentially valuable biomarkers. These varying results may also stem from differences in sample sizes (ranging from Sarabhai et al. *n* = 8 to Pasciato et al. *n* = 88), limiting statistical power.

While Nguyen et al.'s systematic review of FDG PET/MRI in pelvic malignancy identified improved diagnostic accuracy in staging compared to PET/CT, we present multiple studies reporting prognostic imaging parameters in cervical cancer. Despite these limitations, new evidence has emerged in cervical cancer prognostication since the review of Nguyen et al.

### 4.9 Future Indications

There remains heterogeneity in the predictive value of PET/MRI biomarkers for treatment response, prognosis, and metastatic potential. While changes in PET/MRI parameters may correlate with response, their limited ability to guide early treatment modifications reduces their clinical utility. Pre‐treatment SUVmax and ADC values alone have exhibited inconsistent predictive power, possibly due to methodological differences and sample size. Emerging evidence suggests that composite markers, such as TLG, MTV, and HF, may provide superior predictive accuracy. Future studies should focus on standardising imaging protocols, integrating multimodal imaging data, and validating predictive models in larger, prospective trials.

Alongside advanced modelling, a more refined approach to data analysis is essential, especially concerning thresholds. The arbitrary division of continuous imaging data should be avoided, as it leads to a loss of statistical power and results that heavily rely on the chosen, frequently non‐generalisable cut‐off. Instead, future research should favour analysing imaging parameters as continuous variables or employing data‐driven techniques to define clinically relevant thresholds. This approach will strengthen the findings.

Artificial intelligence, including radiomics and machine learning, extracts high‐dimensional quantitative features from medical images. Machine learning algorithms can be trained on these datasets to identify complex patterns linked to clinical endpoints. This approach has the potential for developing highly accurate, individualised prognostic and predictive models, paving the way for personalised cancer care.

## 5 Conclusion

The present study reviews the evidence on correlations between FDG PET and MRI functional and anatomical imaging parameters for evaluating cervical cancer tumours. This study highlights the negative correlation of SUV and ADC values reported by several centres, reflecting tumour biology. We further emphasise the utility of SUV, volumetric metabolic measurements, and MRI ADC values, as well as SUV/ADC correlation, as potential prognostic imaging parameters for locally advanced cervical cancer requiring concomitant chemoradiotherapy.

## Conflicts of Interest

The authors declare no conflicts of interest.

## Supporting information


**Appendix A** Full Electronic Search Strategies.

**Table S1:** QUIPS analysis of included studies.

## Data Availability

Data sharing not applicable to this article as no datasets were generated or analysed during the current study.
