# Supplementary material for: Prognostic Value of Combined FDG PET and MRI Analysis of Cervical Cancer: A Systematic Review and Meta‐Analysis
Source: J Med Radiat Sci. 2026 Jun 18:10.1002/jmrs.70103. Online ahead of print. doi: 10.1002/jmrs.70103 (PMC13399069; doi:10.1002/jmrs.70103)
Supplement: Supplementary file 1 — Appendix A Full Electronic Search Strategies. [file JMRS-9999-0-s001.docx]

# Appendix A. Full Electronic Search Strategies

**Ovid MEDLINE (Ovid) — single line**

(exp Uterine Cervical Neoplasms/ OR (cervical adj3 (cancer* OR carcinoma* OR neoplasm*)).tw,kf.) AND ((PET OR FDG).tw,kf. AND (SUVmax OR "standard uptake value").tw,kf.) AND ((DWI OR "diffusion-weighted").tw,kf. AND (ADC OR ADCmin OR "apparent diffusion coefficient").tw,kf.) AND (correlat* OR associat* OR prognos* OR surviv* OR outcome* OR response OR recur* OR progression).tw,kf.

**Embase (Ovid) — single line**

(exp uterine cervix cancer/ OR (cervical adj3 (cancer* OR carcinoma* OR neoplasm*)).tw,kw.) AND ((PET OR FDG).tw,kw. AND (SUVmax OR "standard uptake value").tw,kw.) AND ((DWI OR "diffusion-weighted").tw,kw. AND (ADC OR ADCmin OR "apparent diffusion coefficient").tw,kw.) AND (correlat* OR associat* OR prognos* OR surviv* OR outcome* OR response OR recur* OR progression).tw,kw.

**Cochrane Library (CENTRAL) — single line**

((cervical NEAR/3 (cancer* OR carcinoma* OR neoplasm*)):ti,ab,kw) AND (((PET OR FDG) AND (SUVmax OR "standard uptake value")):ti,ab,kw) AND (((DWI OR "diffusion-weighted") AND (ADC OR ADCmin OR "apparent diffusion coefficient")):ti,ab,kw) AND ((correlat* OR associat* OR prognos* OR surviv* OR outcome* OR response OR recur* OR progression):ti,ab,kw)*
