# Supplementary material for: Prognostic Value of Combined FDG PET and MRI Analysis of Cervical Cancer: A Systematic Review and Meta‐Analysis
Source: J Med Radiat Sci. 2026 Jun 18:10.1002/jmrs.70103. Online ahead of print. doi: 10.1002/jmrs.70103 (PMC13399069; doi:10.1002/jmrs.70103)
Supplement: Supplementary file 2 — Table S1: QUIPS analysis of included studies. [file JMRS-9999-0-s002.docx]

# Supplementary Information Table 1 – QUIPS analysis of included studies

| **Year** | **Study** | **Participation** | **Attrition** | **Prognostic Factor measurement** | **Outcome measurement** | **Confounding** | **Statistics / reporting** | **Overall** |
| --- | --- | --- | --- | --- | --- | --- | --- | --- |
| 2024 | Dhesi et al. [25] | Moderate | Low -Moderate | Moderate | Moderate | Moderate - High | Moderate | **Moderate** |
| 2023 | Pasciuto et al. [26] | Low - Moderate | Moderate | Low - Moderate | Low | Moderate | Moderate | **Moderate** |
| 2022 | Esfahani et al. [28] | High | Low | Moderate | Moderate | High | High | **High** |
| 2022 | Skipar et al. [30] | Moderate | Low - Moderate | Moderate | Moderate | Moderate | Moderate | **Moderate** |
| 2021 | Shih et al. [29] | Moderate | Low - Moderate | Low | Moderate | Moderate | Moderate - High | **Moderate** |
| 2021 | Steiner et al. [30] | Moderate | Low | Moderate | High | N/A | Moderate | **Moderate - High** |
| 2021 | Vojtisek et al. [31] | Moderate | Moderate | Moderate | Low - Moderate | High | Moderate | **High** |
| 2020 | Gao et al. [33] | Moderate | Moderate | Moderate | Moderate | Moderate | Moderate - High | **Moderate - High** |
| 2019 | Xu Li et al. | Moderate | Moderate | Moderate | Low | Moderate | High | **High** |
| 2019 | Xu Yu et al. | Moderate | Low | Moderate | Low | Moderate | Moderate | **Moderate** |
| 2019 | Du et al. [15] | Moderate - High | High | Moderate | Low - Moderate | Moderate | Moderate - High | **Moderate - High** |
| 2019 | Li-Ou et al. [36] | Moderate | Low | Moderate | N/A | High | Moderate - High | **Moderate - High** |
| 2019 | Akkus Yildirim et al. [11] | Moderate | Low | Moderate | Low - Moderate | Moderate | Moderate | **Moderate** |
| 2019 | Ho et al. [35] | Moderate | Low - Moderate | Low - Moderate | Moderate | Moderate | Moderate | **Moderate** |
| 2019 | Yang et al. [13] | Moderate | High | Moderate | Moderate | High | High |  |
| 2018 | Floberg et al. [14] | High | Low - Moderate | Moderate | Moderate | High | High | **High** |
| 2018 | Sarabhai et al. [39] | High | Moderate | Moderate | Moderate | High | High | **High** |
| 2018 | Kalash et al. [37] | Moderate | High | Moderate | Moderate | Moderate - High | Moderate | **High** |
| 2018 | Mongula et al. [38] | High | Moderate | Moderate | Moderate | High | High | **High** |
| 2018 | Meyer et al. [16] | High | Low | Moderate | N/A | N/A | High | **High** |
| 2017 | Lai et al. [40] | Moderate | Low | Moderate | N/A | N/A | Moderate | **Moderate** |
| 2016 | Pinker et al. [42] | High | High | Moderate | Low - Moderate | High | High | **High** |
| 2015 | Brandmaier et al. [18] | Moderate | Low | Moderate | Low | Low | Moderate | **Moderate** |
| 2015 | Grueneisen et al. [43] | Low - Moderate | Low | Moderate | Moderate - High | High | Moderate | Moderate |
| 2014 | Micco et al. [46] | Moderate | Moderate | Low–Moderate | Moderate | Moderate - High | Moderate - High | **Moderate - High** |
| 2014 | Grueneisen et al. [19] | Moderate | Moderate | Moderate | N/A | N/A | Moderate - High | **Moderate** |
| 2014 | Grueneisen et al. [44] | Moderate | Low | Low | High | N/a | Moderate | Moderate-High |
| 2014 | Kitajima et al. [45] | Moderate | Moderate | Moderate | Moderate | N/A | Moderate | **Moderate** |
| 2014 | Sun et al. [20] | Moderate | Low | Moderate | N/A | N/A | Low - Moderate | **Low - Moderate** |
| 2014 | Surov et al. [17] | Moderate | Low | Moderate | Low - Moderate | High | Moderate - High | **High** |
| 2012 | Nakamura et al. [21] | Moderate | Moderate | Moderate | Low–Moderate | Moderate | Moderate | **Moderate** |
| 2009 | Ho et al. [22] | Moderate | Low | Moderate | N/A | N/A | Moderate | **Moderate** |
| 2017 | Ueno et al. [41] | Moderate | Moderate | Moderate | Moderate | High | Moderate - High | **High** |
| 2019 | Gong et al. [12] | Moderate | Low | Moderate | Low | High | Moderate | **High** |
